# Clinicopathological and Molecular Characterization of Non-Endometrioid Endometrial Carcinoma

**DOI:** 10.7150/jca.108685

**Published:** 2025-03-31

**Authors:** Danqing Zhu, Keyi Shi, Dongxiao Hu, Wanrun Lin, Xiaofei Zhang, Feng Zhou, Yang Li

**Affiliations:** 1 Department of Gynecologic Oncology, Women's Hospital, Zhejiang University School of Medicine, Hangzhou, Zhejiang, China.; 2 Department of Gynecology, Fuyang Women and Children's Hospital, Hangzhou, Zhejiang Province, China, 311400.; 3 Zhejiang Provincial Clinical Research Center for Obstetrics and Gynecology, Hangzhou, Zhejiang, China, 310006.; 4 Laboratory of Pathology, National Cancer Institute, National Institutes of Health, Bethesda, MD, USA, 20892.; 5 Departments of Pathology, The International Peace Maternal and Child Health Hospital, School of Medicine, Shanghai Jiao Tong University, Shanghai, China, 200030.; 6 Shanghai Key Laboratory of Embryo Original Diseases, Shanghai, China, 200030.; 7 Zhejiang Key Laboratory of Maternal and Infant Health, Hangzhou, Zhejiang, China, 310006.

**Keywords:** Non-endometrioid endometrial carcinoma, Molecular classification, Prognosis, Risk factors

## Abstract

**Objective:** Molecular classification has become an essential tool in endometrial carcinoma; however, its application in non-endometrioid carcinoma (NEEC), particularly rare histological subtypes, remains relatively unexplored. This study aims to investigate the potential utility of molecular classification in NEEC.

**Methods**: A retrospective analysis was conducted on 167 NEEC cases diagnosed at the Women's Hospital of Zhejiang University from 2013 to 2020. The cases were categorized into four molecular subtypes: *POLE* ultra-mutated (*POLE*mut), mismatch repair-deficient (MMRd), p53-abnormal (p53abn), and no specific molecular profile (NSMP) molecular subgroups*.* Statistical significance was set at *P*<0.05.

**Results:** Among the cases, 13 (7.8%) patients were classified as *POLE*mut, 25 (15.0%) as MMRd, 84 (50.3%) as p53abn, and 45 (27.0%) as NSMP. Most *POLE*mut cases were at early stages (11/13, 84.6% at stages I-II), whereas p53abn cases were predominantly at advanced stages (32/49, 65.3% at stages III-IV). Additionally, p53abn was the most common subtype in serous carcinoma (41/45, 91.1%) and mixed adenocarcinoma (24/57, 42.1%). The 3-year recurrence-free survival (RFS) rates for *POLE*mut, MMRd, NSMP, and p53abn were 100.0%, 88.0%, 73.3%, and 71.4% , respectively. The 3-year overall survival (OS) rates were 100.0%, 88.0%, 82.2%, and 73.8%, respectively. Univariate analysis revealed significant associations of age ≥60 years (*P*=0.01), hypertension (*P*=0.03), FIGO stage (*P*<0.001), lymphovascular space invasion (*P*=0.01), lymph node metastasis (*P*<0.001), myometrial invasion (*P*<0.001), and postoperative adjuvant therapy (*P*=0.01) with 3-year RFS. Multivariate analysis identified age ≥60 years (*P*=0.03), myometrial invasion (*P*=0.01), and FIGO stage (*P*=0.046) as independent risk factors for 3-year OS.

**Conclusion:** Molecular classification is crucial for accurately predicting the prognosis of NEEC, enabling more tailored treatment approaches in clinical practice. Furthermore, patient age may have a significant influence on NEEC classification and progression.

## Introduction

Endometrial carcinoma (EC) is among the most prevalent gynecological malignancies worldwide, with its incidence rising by a staggering 132% over the past three decades 1. EC is histologically categorized into two main subtypes: endometrioid (Type I) and non-endometrioid (Type II). Endometrioid endometrial carcinoma (EEC), the most common subtype, accounts for approximately 60% of new EC cases but only 25% of EC-related deaths 2. Conversely, non-endometrioid endometrial carcinoma (NEEC) encompasses histological subtypes such as serous, clear cell, carcinosarcoma, dedifferentiated/undifferentiated carcinoma, and mixed carcinoma 3. NEEC, though rare, represents 10%-20% of EC cases yet contributes to 39%-50% of EC-related mortality, underscoring its aggressive nature and the need for more radical treatment strategies 4.

The challenges in managing NEEC are compounded by its histological heterogeneity and the variability in pathologist interpretation, leading to inconsistencies in diagnosis and treatment planning 5. Molecular classification offers an objective, reproducible framework that addresses these challenges by refining diagnostic accuracy and guiding therapeutic decisions. This study seeks to evaluate the clinicopathological characteristics and prognostic implications of molecular subtypes in NEEC. Whereby, it aims to clarify the clinical utility of molecular classification in improving patient outcomes.

## Materials and Methods

### Patients and sample collection

A total of 167 patients pathologically diagnosed as NEEC at Women's Hospital, Zhejiang University School of Medicine, China from January 2013 to December 2020 were enrolled. The inclusion criteria include: (1) Histopathologically confirmed non-endometrioid endometrial carcinoma; (2) Surgical intervention comprising hysterectomy with bilateral salpingo-oophorectomy, with or without pelvic/para-aortic lymphadenectomy and/or omentectomy; (3) Availability of formalin-fixed paraffin-embedded (FFPE) tissue blocks meeting minimum specimen requirements for comprehensive molecular profiling. The exclusion criteria include: (1) Patients submitted to neoadjuvant chemotherapy or hormone therapy before surgery were not included; (2) Patients who had undergone their operations outside of our hospital or those lack follow-up information after surgery were excluded.

Clinicopathological and prognosis data were extracted from the electronic clinical information system database. The last follow-up time was in December 2023. All patients were followed up for at least three years, except for 33 patients who died of disease progression. The 3-year recurrence-free survival (RFS) was defined as the interval between the date of surgery and the date of identified recurrence, and 3-year overall survival (OS) as the interval between the date of surgery and the date of death or the end of follow-up. All the hematoxylin and eosin (H&E)-stained and immunohistochemistry slides were reviewed by senior gynecologic pathologists. All tumors were classified according to the 2020 WHO classification of female genital tumors 4. The study was approved by Ethics Committee of the Hospital (IRB-PRO2022-2436).

### Molecular classification

All samples were classified into four molecular subtypes, *POLE*mut, MMRd, p53abn, or NSMP, based on WHO-endorsed molecular classification 6 DNA was extracted from 5 serial slices with 10-μm-thick for each specimen using NuClean FFPE DNA kit (Catalogue No. CW 2646, China) according to the manufacturer's instructions. PCR-based technology named Dalton-MIT^TM^ (Mutation Identifier Technology) targeting 9 mutation sites within exon 9-14 of *POLE* gene was developed to detect *POLE* mutations 7. MMR or p53 status was determined according to the immunohistochemical (IHC) staining of mismatch repair proteins (MLH1, MSH2, MSH6, and PMS2) and p53 protein. MMRd is defined as the loss of MMR nuclear staining for at least one MMR protein compared with a positive internal control. Mutant p53 staining is defined as a complete loss of nuclear staining in the presence of positive internal control staining (complete absence), strong nuclear expression in over 80% of tumor cells (overexpression), or cytoplasmic staining (cytoplasmic), or a combination of more than one pattern of staining with each present in at least 5% of tumor cells (subclonal mutant expression) 8.

Based on the immunohistochemical and molecular results, cases were classified as follows: "*POLE*mut" if they showed *POLE* mutation; "MMRd" if they showed MMR deficiency in the absence of *POLE* mutations; "p53abn" if they showed p53 aberrant expression in the absence of *POLE* mutations and MMR deficiency; "NSMP" if they were *POLE*-wildtype, MMRp, and p53-wildtype. A molecular group assignment was made in accordance with the TCGA results 9, 10.

### Statistical analysis

The patients' characteristics were described by descriptive statistics. Chi-squared test or Fisher exact test was used for comparison of categorical variables, as appropriate. Survival curves were calculated by the Kaplan-Meier method, and the differences were tested by log-rank test. Simple and multivariable analyses for prognostic factors including RFS and OS were conducted by the Cox proportional hazard model. *P* values < 0.05 were defined as statistically significance. All analyses were performed using the SPSS statistical program version 26.0 (SPSS Inc., Chicago, IL, USA).

## Results

### Clinicopathological features

The main clinical findings are summarized in Table 1. The median age of all patients was 59.0 years, with range from 32 to 90 years. Among 167 enrolled patients, 143 underwent total abdominal hysterectomy with bilateral salpingo-oophorectomy combined with pelvic lymph node dissection, with or without para-aortic lymph node dissection. The remaining 24 patients did not receive comprehensive staging surgery or lymph node dissection. Among them, 94 (56.3%) were stage I, 21 (12.6%) were stage II, 41 (24.6%) were stage III, and 11 (6.6%) were stage IV. Postoperatively, 135 (80.8%) patients received chemotherapy/radiation therapy, 10 (6.0%) patients received progestogen therapy. Histologically, 57 (34.1%) were mixed cell adenocarcinoma, 45 (26.9%) were serous carcinoma, 40 (24.0%) were carcinosarcoma, 11 (6.6%) were clear cell carcinoma, 11 (6.6%) were undifferentiated/dedifferentiated carcinoma, 2 (1.2%) were neuroendocrine carcinoma, 1 (0.6%) was mesonephric adenocarcinoma. All the patients had complete follow-up information available in the present study. Of them, 95 (56.9%) patients were alive without disease, 39 (23.4%) patients relapsed, and 33 (19.8%) patients died after diagnosis. The clinicopathological parameters are listed in Table 1.

### Molecular subtypes in NEEC

All the 167 samples of NEEC were analyzed for molecular classification. Totally, 13 patients (7.8%) were classified as *POLE*mut, 25 (15.0%) patients were classified as MMRd, 84 (50.3%) patients were classified as p53abn and 45 (27.0%) patients were classified as NSMP. In this study, eight (4.8%) cases with dual molecular characteristics were found, of which two cases with *POLE*mut-MMRd were classified as *POLE*mut subtype, and six cases with MMRd-p53abn were classified as MMRd subtype. Of note, 8.8% (5/57) of mixed cell adenocarinoma, 6.7% (3/45) of serous carcinoma, 2.5% (1/40) of carcinosarcoma, 9.1% (1/11) of clear cell carcinoma, and 27.3% (3/11) of undifferentiated/dedifferentiated carcinoma had the *POLE*mut, respectively. While no *POLE*mut was found in neuroendocrine carcinoma (0/2) or mesonephric adenocarcinoma (0/1). Most the subtypes of p53abn were found in serous carcinoma (91.1%) and mixed cell adenocarcinoma (42.1%). The distribution of molecular subtypes is listed in Table 1.

**Figure 1:**
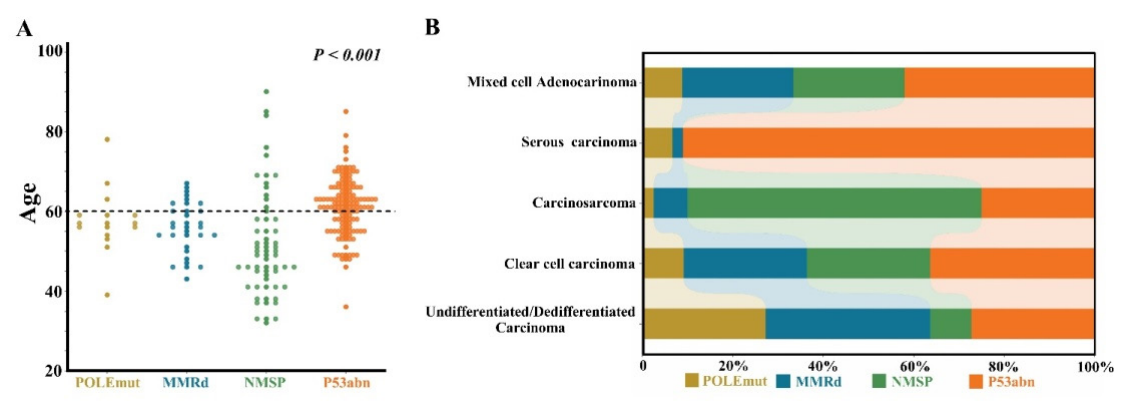
There were significant differences in the age (A) and distribution of pathological types (B) among different molecular subtypes.

The median age of p53abn patients was 61.5 (36-85) years, and 64.3% aged 60 years or over. However, patients with *POLE*mut subtype or NSMP subtype were relatively younger, with mean age of 57.0 (39-78) and 50.0 (32-90) years, respectively. Patients under 60 years old accounted for 76.9% and 71.1%, respectively. There was significant difference among the four subtypes (Figure 1A).

Of note, the majority of *POLE*mut patients were at early stage, with 84.6% (11/13) of patients at stages I-II; while NEEC patients with p53abn were mainly at advanced stages, accounting for 65.3% (32/49) of all patients at stages III-IV. There were significant differences in the distribution of pathological types among different molecular subtypes (P<0.001) (Figure 1B).

### Patients with *POLE*mut NEEC had the best prognosis

**Figure 2:**
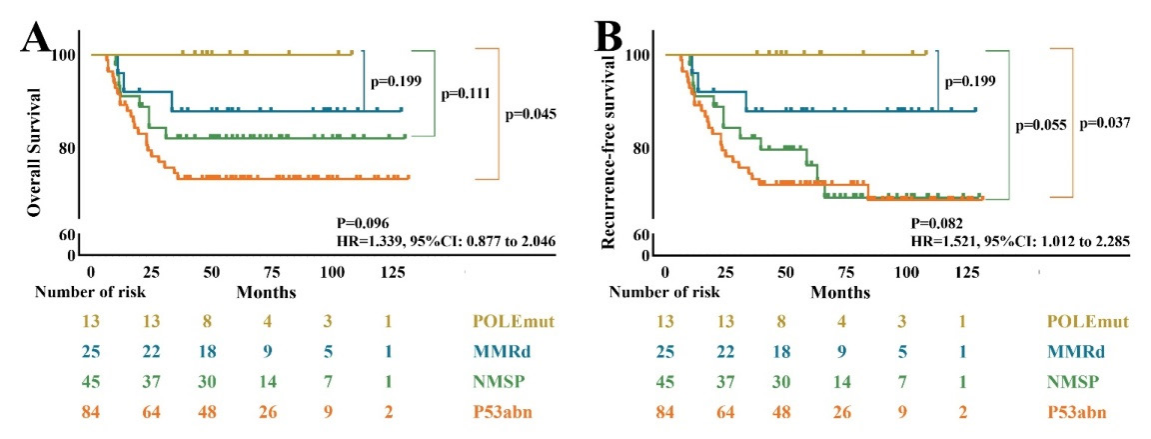
Survival curves for molecular subgroups of non-endometrial endometrial carcinoma. (A) Kaplan-Meier (KM) curves for overall survival (OS); (B) KM curves for recurrence-free survival (RFS).

The average follow-up time was 61.2 ± 2.5 months, with range from 6.5 to 131.5 months. The *POLE*mut patients had the best prognosis, without any recurrence during the follow-up time. The prognosis of p53abn subtype was the worst with 3-year RFS of 71.4% and OS of 73.8%, with median RFS was 53.0 month. NSMP subtype was followed, with 3-year RFS of 73.3% and OS of 82.2%, with median RFS 53.0 month. The 3-year RFS and OS of MMRd subtype were both 88.0%, with median RFS 58.5month (Table 2 and Figure 2).

Univariate survival analysis of prognostic factors by Kaplan-Meier rank analysis showed that age ≥ 60 years (P=0.01), hypertension (P=0.03), stage (P<0.001), LVSI (P=0.01), lymph node metastasis (P<0.001), depth of myometrial invasion (P<0.001), adjuvant treatment (P=0.01) were significantly associated with prognosis (Table 3). A multivariate analysis of these variables was performed using the Cox proportional hazard regression model. It showed that age ≥ 60 years (HR 2.40, 95%CI 1.07 to 5.39, P=0.03), myometrial invasion (HR 3.34, 95%CI 1.31 to 8.53 P=0.01), and FIGO stage (HR 2.92, 95%CI 1.39 to 6.14, P=0.046) as independent risk factors for 3-year OS.

## Discussion

NEEC is relatively rare and consists of a collection of various aggressive histological subtypes of endometrial cancer, which collectively tend to exhibit a poor prognosis due to their aggressive nature. Similar to endometrioid endometrial carcinoma (EEC), several clinicopathological factors, including FIGO stage, LVSI, lymph node metastasis, and myometrial invasion, have been associated with recurrence risk in NEEC 11 12. In our study, myometrial invasion and FIGO stage were identified as independent risk factors for prognosis in NEEC patients. Additionally, age has been previously recognized as a risk factor for recurrence in EEC 13, 14, with the 2024 NCCN guidelines recommending adjuvant management stratification for early-stage EEC based on an age threshold of 60 years. Our findings indicate that age is also a significant factor in NEEC, with patients aged 60 years or older exhibiting a significantly higher risk of recurrence and death. This suggests that age plays a crucial role in the classification and progression of NEEC, warranting further investigation into personalized treatment strategies.

Due to the invasive and metastatic nature of NEEC 15, treatment strategies are often more aggressive than those for EEC. However, whether all NEEC cases require such aggressive approaches remains an open question for gynecological oncologists and pathologists. NEEC poses significant challenges in histological classification due to its heterogeneity and the difficulty of achieving consistent reproducibility with traditional methods. This inconsistency, particularly in assessing morphological risk factors such as grade and LVSI 16, can lead to discrepancies in diagnosis and treatment planning. Molecular classification offers a promising solution to these challenges by providing a more objective and reproducible framework for diagnosis, treatment, and prognosis.

Despite its promise, molecular classification has limitations, particularly concerning the inclusion of specific histological subtypes. The TCGA classification, based on 373 endometrial carcinoma cases, predominantly focused on EEC (82.3%), with limited representation of serous (n=53, 14.2%) and mixed subtypes (n=13, 3.5%) 2. Data on other rare histological subtypes, such as undifferentiated carcinoma, remain scarce. Similarly, the TransPORTEC classification, which analyzed high-risk endometrial cancer cases, included only 25.9% NEEC 17. These differences in cohort composition highlight the distinct molecular subtype distributions within various pathological subtypes. For example, Travaglino et al. found that microsatellite instability (MSI) was the dominant molecular subtype (44%) in undifferentiated/dedifferentiated endometrial cancer 18. In contrast, their analysis of 162 endometrial clear cell carcinoma cases from five studies revealed that high-copy (42.5%) and low-copy (40.9%) subtypes were most prevalent, while MSI (9.8%) and *POLE*mut (3.8%) were less common 19. This underscores the molecular diversity within clear cell carcinomas.

Our study analyzed NEEC molecular subtypes, focusing on mixed cell adenocarcinoma, serous carcinoma, and carcinosarcoma, while excluding EEC to objectively assess the value of molecular classification in NEEC. Among mixed cell adenocarcinomas, 42.1% were p53abn, while serous carcinomas exhibited an even higher prevalence of p53abn (91.1%), consistent with previous reports 20. In carcinosarcomas, NSMP was the most common subtype (65.0%), whereas among clear cell carcinomas, *POLE*mut was the least common (9.1%), with p53abn being the most frequent (36.4%). These findings provide valuable insights into the molecular subtype distribution across different NEEC histological types. Of note, p53 status was determined according to the IHC staining of mismatch repair proteins and p53 protein in the present study. There is a possibility of *POLE*mut-p53abn and *POLE*mut- p53abn, MMRd-p53abn subtypes in the queue of serous cancers diagnosed solely based on pathological morphology and histology. These patients will suffer from excessive treatment and lose the opportunity for immunotherapy.

The *POLE*mut subtype is associated with an excellent prognosis 21. Large meta-analyses indicate that most *POLE*mut cases are early-stage (FIGO I-II: 88%, 92%), endometrioid in histology (88.3%, 84.5%), without lymph node metastasis (66.2%, 74.3%), and frequently lack LVSI (66.2%, 77.7%) 22, 23. The 2021 ESGO guidelines 24 and ongoing clinical trials (e.g., PORTEC-4a 25, PROBEAT 6, and RAINBOW 26) recommend de-escalation or no adjuvant therapy for *POLE*mut patients. However, these recommendations primarily target EEC, leaving a gap in data regarding *POLE*mut NEEC. In our study, *POLE*mut NEEC accounted for 7.8% of cases, similar to the TCGA database. Despite their high malignancy, *POLE*mut NEEC shares clinicopathological and prognostic similarities with EEC, suggesting that their underlying molecular mechanisms may be consistent. Prospective studies are essential to validate these findings.

p53abn, the molecular subtype with the poorest prognosis, is most prevalent in serous carcinoma and poorly differentiated endometrioid carcinoma, as well as other tissue types and low-grade tumors 3, 26. Our findings show that p53abn was present in 91.1% of serous carcinomas, 42.1% of mixed cell adenocarcinomas, and 25% of carcinosarcomas. Most p53abn NEEC cases were at advanced stages (60.8%, stages III-IV), with a significant proportion of patients aged 60 or older (64.3%). This aligns with previous research linking advanced age to poor NEEC prognosis 27, 28. Conversely, MMRd and NSMP subtypes exhibited intermediate prognoses without distinct clinical or pathological features. Ongoing clinical trials aim to identify more effective treatments to improve outcomes for these subtypes.

Overall, this study highlights the significant differences in patient age and histological type distributions across the four molecular subtypes of NEEC. Molecular classification provides valuable insights into the clinical and pathological characteristics of NEEC, reinforcing its importance in the precise diagnosis and treatment planning of endometrial cancer. Future high-quality studies are needed to further validate and refine the clinical applications of molecular classification in NEEC.

## Figures and Tables

**Table 1:** Clinicopathological Features of Molecular Subgroup in Non-endometrioid endometrial carcinoma

Variable	N=167		Subtypes				*P*-value
		*POLE*mut (n=13)	MMRd (n=25)	p53abn (n=84)	NSMP (n=45)		*P*-value
**Age, y**							***P*<0.001**
<60	88 (52.7%)	10 (76.9%)	16 (64.0%)	30 (35.7%)	32 (71.1%)		
≥60	79 (47.3%)	3 (23.1%)	9 (36.0%)	54 (64.3%)	13 (28.9%)		
**BMI (kg/m^2^)**							
<25	111 (66.5%)	8 (61.5%)	16 (64.0%)	56 (66.7%)	31 (68.9%)		*P*=0.96
≥25	56 (33.5%)	5 (38.5%)	9 (36.0%)	28 (33.3%)	14 (31.1%)		
**Hypertension**							*P*=0.33
Yes	55 (32.9%)	6 (46.2%)	7 (28.0%)	31 (36.9%)	11 (24.4%)		
NO	112 (67.1%)	7 (53.8%)	18 (72.0%)	53 (63.1%)	34 (75.6%)		
**Diabetes**							*P*=0.54
Yes	19 (11.4%)	1 (7.7%)	5 (20.0%)	9 (10.7%)	4 (8.9%)		
NO	148 (88.1%)	12 (92.3%)	20 (80.0%)	75 (89.3%)	41 (91.1%)		
**Hormone therapy**							*P*=0.57
Yes	10 (6.0%)	1 (7.7%)	1 (4.0%)	7 (8.3%)	1 (2.2%)		
No	157 (94.0%)	12 (92.3%)	24 (96.0%)	77 (91.7%)	44 (97.8%)		
**FIGO Stage**							*P*=0.36
I	94 (56.3%)	9 (69.2%)	15 (60.0%)	46 (54.8%)	24 (53.3%)		
II	21(12.6%)	2 (15.4%)	5 (20.0%)	6 (7.1%)	8 (17.8%)		
III	41(24.6%)	2 (15.4%)	5 (20.0%)	25 (29.8%)	9 (20.0%)		
IV	11(6.6%)	0 (0%)	0 (0%)	7 (8.3%)	4 (8.9%)		
**Pregnancy history**							*P*=0.54
≤3	122 (73.1%)	10 (76.9%)	17 (68.0%)	65 (77.4%)	30 (66.7%)		
>3	45 (26.9%)	3 (23.1%)	8 (32.0%)	19 (22.6%)	15 (33.3%)		
**Family history of cancer**							*P*=0.84
Yes	17 (10.2%)	2 (15.4%)	3 (12.0%)	8 (9.5%)	4 (8.9%)		
NO	150 (89.8%)	11 (84.6%)	22 (88.0%)	76 (90.5%)	41 (91.1%)		
**Histology**							***P*<0.001**
Mixed cell Adenocarinoma	57 (34.1%)	5 (38.5%)	14 (56.0%)	24 (28.6%)	14 (31.1%)		
Serous carcinoma	45 (26.9%)	3 (23.1%)	1 (4.0%)	41 (48.8%)	0 (0.0%)		
Carcinosarcoma	40 (24.0%)	1 (7.7%)	3 (12.0%)	10 (11.9%)	26 (57.8%)		
Clear cell carcinoma	11 (6.6%)	1 (7.7%)	3 (12.0%)	4 (4.8%)	3 (6.7%)		
Undifferentiated/ Dedifferentiated Carcinoma	11 (6.6%)	3 (23.1%)	4 (16.0%)	3 (3.6%)	1 (2.2%)		
Neuroendocrine carcinoma	2 (1.2%)	0 (0.00%)	0 (0.0%)	2 (2.4%)	0 (0.00%)		
Mesonephric adenocarcinoma	1 (0.6%)	0 (0.00%)	0 (0.0%)	0 (0.0%)	1 (2.2%)		
**LVSI**							*P*=0.76
Yes	46 (27.5%)	2 (15.4%)	7 (28.0%)	23 (27.4%)	14 (31.1%)		
No	121 (72.6%)	11 (84.6%)	18 (72.0%)	61 (72.6%)	31 (68.9%)		
**Lymph node status**							*P*=0.30
Positive	33 (19.8%)	1 (7.7%)	3 (12.0%)	21 (25.0%)	8 (17.8%)		
Negative	110 (65.9%)	11 (84.7)	20 (80.0%)	59 (70.2%)	20 (44.4%)		
Not available	24 (14.3%)	/	/	/	/		
**Myometrial invasion**							*P*=0.30
<50%	78 (46.7%)	9 (69.2%)	16 (64.0%)	36 (42.9%)	17 (37.8%)		
≥50%	56 (33.5%)	3 (23.1%)	6 (24.0%)	30 (35.7%)	17 (37.8%)		
Confined to the inner membrane	33 (19.8%)	1(7.7%)	3(12.0%)	18 (21.4%)	11 (24.4%)		

**Table 2:** Distribution of recurrence and death of each molecular subtype in patients with non-endometrioid endometrial cancer

Molecular typing	Total	Number of relapses	RFS	*P*-value	Number of deaths	OS	P-value
*POLE*mut	13	0	100%	P=0.05	0	0.0%	P=0.09
MMRd	25	3	88.0%	P=0.05	3	88.0%	P=0.09
p53abn	84	24	71.4%	P=0.05	22	73.8%	P=0.09
NSMP	45	12	73.3%	P=0.05	8	82.2%	P=0.09

**Table 3:** Univariate and multivariate analyses affecting recurrence-free survival in patients with non-endometrioid endometrial cancer

Variable	N=167	Univariable analysis	Univariable analysis	Univariable analysis	Multivariable analysis	Multivariable analysis	Multivariable analysis
		HR	95%CI	*P*-value	HR	95%CI	*P*-value
**Age, y**							
<60	88	1			1		
≥60	79	2.33	1.21 to 4.48	*P*=0.01	1.92	0.91 to 4.09	*P*=0.09
**BMI (kg/m^2^)**							
<25	111	1			-		
≥25	56	0.99	0.51 to 1.94	*P*=0.99			
**Hypertension**							
NO	112	1			1		
Yes	55	2.02	1.07 to 3.79	*P*=0.03	1.88	0.96 to 3.68	*P*=0.07
**Diabetes**					-		
Yes	19	1					
NO	148	0.77	0.30 to 1.98	*P*=0.59			
**Hormone therapy**					-		
Yes	10	1					
No	157	1.08	0.26 to 4.50	*P*=0.91			
**Pregnancy history**					-		
≤3	122	1					
>3	45	0.88	0.41 to 1.81	*P*=0.73			
**Family history of cancer**					-		
Yes	17	1					
NO	150	5.45	0.75 to 39.74	*P*=0.10			
**FIGO Stage**				*P*<0.001			*P*=0.04
I	94	1			1		
II	21	4.89	1.41 to16.88	*P*=0.01	2.34	0.59 to 9.32	*P*=0.23
III	41	14.41	5.46 to 38.05	*P*<0.001	4.15	1.14 to 15.11	*P*=0.03
IV	11	15.50	4.72 to 50.88	*P*<0.001	7.30	1.81 to 29.41	*P*=0.01
**Histology**				*P*=0.209			
Serous carcinoma	45	1					
Mixed cell adenocarinoma	57	1.02	0.49 to 2.10	*P*=0.97			
Carcinosarcoma	40	0.30	0.10 to 0.93	*P*=0.04			
Clear cell carcinoma	11	0.58	0.13 to 2.55	*P*=0.47			
Undifferentiated/ Dedifferentiated Carcinoma	11	0.72	0.16 to 3.21	*P*=0.67			
Neuroendocrine carcinoma	2	-	-	*P*=0.28			
Mesonephric adenocarcinoma	1	-	-	*P*=0.98			
**LVSI**				*P*=0.01			
NO	121	1			1		
Yes	46	2.37	1.25 to 4.49	*P*=0.01	1.76	0.77 to 4.03	*P*=0.18
**Lymph node status**				*P*<0.001			
Negative	33	1			1		
Positive	110	7.6	3.88 to14.91	*P*<0.001	2.12	0.77 to 5.88	*P=*0.15
Not available	24	*/*	*/*	*/*	*/*	*/*	*/*
**Myometrial invasion**				*P*<0.001			*P*=0.004
≥50%	56	1			1		
<50%	111	0.17	0.09 to 0.34	*P*<0.001	3.79	1.51to 9.54	*P*=0.01
**Adjuvant treatment**				*P*=0.013			*P*=0.71
None	22	1			1		
Chemotherapy	38	3.97	0.49 to 32.29	*P*=0.20	0.33	0.03 to 3.26	*P*=0.34
Radiotherapy	22	4.24	0.47 to 37.94	*P*=0.20	0.96	0.32 to 2.92	*P*=0.94
Radiochemotherapy	75	9.13	1.24 to 67.34	*P*=0.03	1.23	0.15 to 1.81	*P*=0.88
Progestogen	10	1.93	0.12 to 30.94	*P*=0.64	0.56	0.08 to 18.84	*P*=0.53
**Molecular typing**				*P*=0.08	-		
p53abn	84	1					
*POLE*mut	13	-	-	*P*=0.97			
MMRd	25	0.38	0.11 to 1.26	*P*=0.11			
NSMP	45	0.87	0.43 to 1.74	*P*=0.69			

**Table 4:** Univariate and multivariate analysis of overall survival in patients with NEEC

Variable	N=167	Univariable analysis	Univariable analysis	Univariable analysis	Multivariable analysis	Multivariable analysis	Multivariable analysis
		HR	95%CI	*P*	HR	95%CI	*P*
**Age, y**							
<60	88	1			1		
≥60	79	2.92	1.39 to 6.14	*P*=0.01	2.40	1.07 to 5.39	*P=*0.03
**BMI (kg/m^2^)**							
<25	111	1					
≥25	56	1.30	0.65 to 2.61	*P*=0.46			
**Hypertension**							
NO	112	1			1		
Yes	55	2.75	1.39 to 5.47	*P*=0.004	2.13	1.04 to 4.37	*P*=0.04
**Diabetes**							
Yes	19	1					
NO	148	0.65	0.25 to 1.69	*P*=0.38			
**Pregnancy history**							
≤3	122	1					
>3	45	0.83	0.37 to 1.83	*P*=0.64			
**Family history of cancer**							
Yes	17	1			-		
No	150	24.22	0.22 to 2636.26	*P*=0.04	-	-	*P*=0.98
**Hormone therapy**							
Yes	10	1					
No	157	0.94	0.23 to 3.93	*P*=0.93			
**FIGO Stage**				*P*<0.001			*P*=0.046
I	94	1			1		
II	21	5.92	1.59 to 22.04	*P*=0.01	2.80	0.71 to 10.99	*P*=0.14
III	41	13.55	4.60 to 39.88	*P*<0.001	2.88	0.65 to 12.81	*P*=0.17
IV	11	15.74	4.22 to 58.72	*P*<0.001	7.71	1.76 to 33.73	*P*=0.01
**Histology**				*P*=0.12			
Serous carcinoma	45	1					
Mixed cell Adenocarcinoma	57	0.81	0.38 to 1.75	*P*=0.60			
Carcinosarcoma	40	0.16	0.04 to 0.70	*P*=0.02			
Clear cell carcinoma	11	0.62	0.14 to 2.75	*P*=0.53			
Undifferentiated / Dedifferentiated Carcinoma	11	0.69	0.16 to 3.05	*P*=0.62			
Neuroendocrine carcinoma	2	2.84	0.37 to 21.71	*P*=0.32			
Mesonephric adenocarcinoma	1	-	-	*P*=0.98			
**LVSI**				*P*=0.004			
No	121	1			1		
Yes	46	2.64	1.34 to 5.23	*P*=0.01	1.17	0.50 to 2.73	P=0.73
**Lymph node status**				*P*<0.001			
Negative	110	1			1		
Positive	33	7.29	3.53 to 15.06	*P*<0.001	2.51	0.85 to 7.40	*P*=0.09
Not available	24	*/*	*/*	*/*	*/*	*/*	*/*
**Myometrial invasion**				*P*<0.001			*P*=0.01
<50%	111	1			1		
≥50%	56	6.75	3.13 to 14.54	*P*<0.001	3.34	1.31 to 8.53	*P*=0.01
**Adjuvant treatment**				*P*=0.19			
None	22	1					
Chemotherapy	38	2.88	0.34 to 24.61	*P*=0.34			
Radiotherapy	22	5.53	0.62 to 49.46	*P*=0.13			
Radiochemotherapy	75	7.70	1.04 to 57.06	*P*=0.046			
Progestogen	10	-	-	*P*=0.98			
**Molecular typing**				*P*=0.09			
p53abn	84	1					
*POLE*mut	13	-	-	*P*=0.974			
MMRd	25	0.42	0.13 to 1.40	*P*=0.156			
NSMP	45	0.64	0.28 to 1.43	*P*=0.271			
